# IS THERE A CLINICAL PATHOLOGICAL CORRELATION OF COLORECTAL ADENOCARCINOMA WITH THE IMMUNOHISTOCHEMICAL EXPRESSION OF OPN AND ABCB5?

**DOI:** 10.1590/0102-672020200004e1569

**Published:** 2021-03-23

**Authors:** Diogo Francesco CASTOLDI, Osvaldo MALAFAIA, Pedro Helo dos SANTOS-NETO, Tatiana Varella POSTIGLIONI, Cecilia VASCONCELOS, Fabiola Past BREMER, Leticia Elizabeth Augustin CZECZKO, Martin GASSER, Ana Maria WAAGA-GASSER, Carmen Australia Paredes Marcondes RIBAS

**Affiliations:** 1 Medical Research Institute, Mackenzie Evangelical School of Paraná, Curitiba, PR, Brazil; 2 Mackenzie Evangelical School of Paraná, Curitiba, PR, Brazil; 3 Department of Surgery, Mackenzie Evangelical School of Paraná, Curitiba, PR, Brazil; 4 Department of Hematology, Mackenzie Evangelical School of Paraná, Curitiba, PR, Brazil; 5 Department of Oncology, Mackenzie Evangelical School of Paraná, Curitiba, PR, Brazil; 6 Department of Surgery, University Hospital Würzburg, Würzburg, Germany; 7 Renal Division, Brigham and Woman’s Hospital, Harvard Medical School, Boston, MA, USA; 8 Evangelical Mackenzie University Hospital, Curitiba, Paraná, Brazil

**Keywords:** ABCB5, Osteopontin, Colorectal cancer, Tumor biomarkers, ABCB5, Osteopontina, Câncer colorretal, Biomarcadores tumorais

## Abstract

**Background:**

Studies with biomarkers in TMA (tissue microarray) have been showing important results regarding its expression in colon cancer.

**Aim:**

Correlate the expression profile of the OPN and ABCB5 biomarkers with the epidemiological and clinicopathological characteristics of the patients, the impact on the progression of the disease and the death.

**Method:**

A total of 122 CRC patients who underwent surgical resection, immunomarking and their relationship with progression and death events were evaluated.

**Result:**

The average age was 61.9 (±13.4) years. The cases were distributed in 42 (35.9%) in the ascending/transverse colon, 31 (26.5%) in the sigmoid, 27 in the rectum (23.1%), 17 (14.5%) in the descending colon. Most patients had advanced disease (stages III and IV) in 74 cases (60.9%). There was a predominance of moderately differentiated tumors in 101 samples (82.8%); despite this, the poorly differentiated subtype proved to be an independent risk factor for death in 70%. Metastasis to the liver proved to be an independent risk factor for death in 75% (18/24), as well as patients with primary rectal tumors in 81.5% (22/27).

**Conclusion:**

The immunohistochemical expression of the OPN and ABCB5 markers was not associated with epidemiological and clinicopathological characteristics. Regarding the progression of disease and death, it was not possible to observe a correspondence relationship with the evaluated markers.

## Graphical abstract

[Fig f1a]

**Figure f1a:**
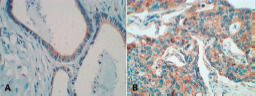
OPN ABCB5

[Fig f1as]

**OPN ABCB5 f1as:**
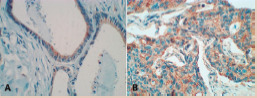

## RESUMO

**Racional:**

Estudos com biomarcadores com TMA (tissue microarray) vêm demostrando resultados importantes em relação à expressão de biomarcadores em câncer de cólon.

**Objetivo:**

Correlacionar o perfil de expressão dos biomarcadores OPN e ABCB5 com as características epidemiológicas e clinicopatológicas dos pacientes, o impacto na progressão de doença e no evento óbito.

**Método:**

Foram avaliados 122 pacientes de CCR submetidos à ressecção cirúrgica e à imunomarcação e relação com os eventos progressão e óbito.

**Resultado:**

A média de idade encontrada foi de 61,9 (±13,4) anos. Os casos distribuíram-se em 42 (35,9%) no cólon ascendente/transverso, 31 (26,5%) no sigmoide, 27 no reto (23,1%), 17 (14,5%) no cólon descendente. A maioria dos pacientes apresentou doença avançada (estadio III e IV) em 74 casos (60,9%). Houve predomínio de tumor moderadamente diferenciado em 101 amostras (82,8%); apesar disso, o subtipo pouco diferenciado mostrou-se como fator de risco independente para óbito em 70% dos casos. Metástase para o fígado mostrou-se fator de risco independente para óbito em 75% dos casos (18/24), assim como pacientes com tumores primários de reto em 81,5% (22/27).

**Conclusão:**

A expressão imunoistoquímica dos marcadores OPN e ABCB5 não apresentou associação com as características epidemiológicas e clinicopatológicas. Em relação à progressão de doença e evento óbito, não se conseguiu observar relação de correspondência com os marcadores avaliados.

## INTRODUCTION

Colorectal cancer (CRC) is the third most diagnosed malignancy in the world, considered one of the main causes of death^15^ ^,^ ^22^ ^,^ ^32^. Treatment consists of surgery, chemotherapy and radiation therapy. However, despite advances in treatment, the prognosis in cases of metastatic tumors remains reserved^32^ ^,^ ^11^. 

Colorectal polyps are considered in most cases to be the precursors of RCC, according to the adenoma-adenocarcinoma progression sequence, with variation in the speed of progression according to the characteristic of each polyp^30^. When not early diagnosed, and when operated on in emergency situations, it may present with major hydroelectrolytic changes, worsening the global prognose^8^. 

Regarding molecular biology, there is great diversity, among which stand out the microsatellite instability, mutations in DNA repair genes and genetic changes such as APC, TP53, SMAD4, PIK3CA, KRAS, SOX9^19^, just to name a few genes. 

Minimally invasive blood markers have gained importance due to the facility for screening and monitoring patients^3^ ^,^ ^4^ ^,^ ^14^. The carcinoembryonic antigen (CEA) is the most used in CCR, however its serum value may suggest, but not confirm the diagnosis or metastasis of cancer^20^ ^,^ ^14^. 

**FIGURE 1:**
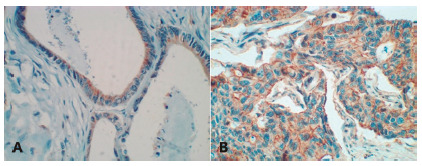
Colon adenocarcinoma: A) OPN showing cytoplasmic positivity and in brown the region of its marking on the cytoplasmic membrane is observed; B) ABCB5 showing positivity of the cytoplasmic membrane and in brown the region of its marking inside and cytoplasmic membrane (400x) is observed

There are few studies with biomarkers in microarray tissue blocks (TMA) in the literature; however, they have been showing significant results, as occurs in the expression of osteoponin (OPN): weak in healthy colonic cells, moderate in adenomas and strong in colon cancer^24^. OPN (Figure 1A) is an extracellular, multifunctional glycophosphoprotein, found in mineralized tissues such as extracellular matrix (ECM), body tissues and fluids, including blood, milk, urine, saliva, seminal fluid and bile^32^ ^,^ ^25^ ^,^ ^17^ ^,^ ^28^. It is produced in five isoforms by endothelial, neural cells, macrophages, monocytes, T lymphocytes, responsible for the regulation of immune response, cell regeneration, vascularization, migration and motility^1^ ^,^ ^2^ ^,^ ^26^. It belongs to the Sibling family (Small Integrin-Binding Ligand N-linked Glycoprotein), a group of proteins located on chromosome 4, which have been observed in different tumor stages, indicating the potential for modulating the early tumoral behavior^7^. 

The expression of OPN is regulated by stimuli and pathways associated with CCR progression and metastasis. When derived from host cells, it induces cellular immunity and can reinforce anti-tumor protection by cytotoxic T lymphocytes. When elevated, it can promote tumor progression and cell survival, neoangiogenesis and metastasis. This high induced expression of OPN in normal cells is observed to induce behavior similar to that of neoplastic cells, including cell invasion in vitro and metastasis in vivo^25^.

The ABC genes constitute the largest family of transmembrane proteins, whose function is the transport of molecules with consumption of ATP^6^. The ABCB5 gene (Figure 1B), belongs to the superfamily ABC^31^, located on chromosome 7, short arm, loci 14^6^, has varied expression in tissues such as melanocytes, pigmented epithelium of the retina and breast^12^, and increased expression in breast, colorectal and melanoma cancer malignant^12^ ^,^ ^9^ ^,^ ^23^. Most ABC genes transport substances from the cytoplasm to the cell exterior or to intracellular compartments^6^, through the drug efflux mechanism, presenting a protective effect of cancer stem cells against the attack of chemotherapeutic agents, for example.

North American data show a five-year survival rate of 88.1% and 12.6% for stages I and IV of the disease, respectively^5^. Considering the above, the evaluation of the expression by immunohistochemistry of OPN and ABCB5 proteins in tissue using CCR TMA can help in a better prognosis and early detection of these tumors. Diagnostic and therapeutic strategies are necessary, which requires a greater understanding of the molecular mechanisms of RCC and the use of biomarkers in order to improve the prognosis through the early detection of these tumors^11^ ^,^ ^20^.

The aim of this study was to correlate the expression profile of the OPN and ABCB5 biomarkers with the epidemiological and clinicopathological characteristics of the patients, the impact on disease progression and on the death event.

## METHODS

This is an observational, retrospective and single-center analytical study, of the case-control type. The research was previously approved by the Research Ethics Committee of Faculdade Evangélica Mackenzie do Paraná under no. 1,999,670.

### Patients and tissues

Patients were selected from Hospital Universitário Evangélico Mackenzie in Curitiba, PR, Brazil, of both genders, over 18 years old, with a diagnosis of colorectal adenocarcinoma, were seen and treated during the period of 2010 and 2015, whose paraffin blocks confirmed the diagnosis. These blocks should be eligible for further fractionation for evaluation by TMA, and the medical record should be available at the hospital. These blocks and slides were sent for histological confirmation by a second pathologist and were subsequently sent for immunostaining.

The exclusion criteria were: age under 18; diagnosis of colorectal cancer not adenocarcinoma; paraffin blocks could not be found or manipulated; absence of clinical and epidemiological data in medical records.

The application of the inclusion and exclusion criteria resulted in 122 patients, this being the final sample of the study.

### Defining groups

From the data collection, patients were divided into three distinct groups, associated with the OPN and ABCB5 markers as negative (control), positive (case 1) and inconclusive (case 2) for their expression. From this division, patients were also subdivided into patients with local and advanced disease.

### Block making and immunohistochemistry

The Tissue Tek Quick-ArrayTM manual device was used to make the multi-sample blocks, which contains coupled clamps whose diameters vary from 1.0 mm to 3.0 mm, responsible for extracting the desired area for the immunohistochemistry. These blocks made it possible to obtain up to 60 fragments of neoplastic tumor tissue. From this point on, they were submitted to the immunoperoxidase technique, performed on a Benchmark UltraTM instrument, with integrated 3 in 1 processing, including dewaxing, rehydration and antigenic recovery, with Cell Conditioning 1 (high pH) and Cell Conditioning 2 (low pH) buffers. ). The incubation with antibodies of the ABCB5 marker (clone 5H3C6 manufacturer Genetex) in the 1: 100 dilution and the OPN marker (polyclonal, manufacturer Medaysis) in the 1:20 dilution, lasted between 16-20 min at room temperature. The amplification was performed by Ultraview Universal DAB Detection Kit. All processing was carried out on a Ventana Benchmark UltraTM automated platform. Positive internal and external controls attested the fidelity of the reactions. After marking the primary antibodies, the reading was done through an amplifier. Samples that showed labeling by the antibody were considered positive and negative those that were not marked.

### Data collect

After selecting the cases, clinical information was collected. For this purpose, the following databases were used: electronic medical record system (PAGU), physical ambulatory medical record, chemotherapy release guides (APACs, when available) and official pathological report of colorectal tumors. Telephone contact was followed for additional information, when possible. The data were distributed in an Excel table according to the following standardized protocol: age, gender, date of diagnosis, report of pathological anatomy, staging, treatments used (surgery, chemotherapy and / or radiotherapy), presence and location of lymph node or distant metastases, existence of disease progression, date of last visit and / or death.

### Statistical analysis

The data were analyzed using the computer program Stata / SE v.14.1. StataCorpLP, USA. Quantitative variables were described as means, standard deviations, medians, minimum and maximum values. Categorical variables were described by frequencies and percentages. Fine and Gray models were adjusted to analyze factors related to the time until disease progression (Pevent) considering death as a competitive risk. After adjustment, the estimated association measure was the subdistribution hazard ratio (SHR). For the survival analysis, Cox regression models were adjusted, and the hazard ratio values ​​were estimated. For both models, the Wald test was used to assess the significance of the variables. Values ​​of p <0.05 indicated statistical significance. 

## RESULTS

The average age found was 61.9± 13.4 years. In the total sample, there was a male / female ratio of 1.06: 1, with a higher prevalence of disease in the fifth and sixth decade of life (56.6%). The cancer incidence according to the topography was 42 cases (35.9%) in the ascending / transverse colon, 31 (26.5%) in the sigmoid, 27 in the rectum (23.1%), 17 (14.5 %) in the descending colon and five indeterminate. Regarding the degree of differentiation, there was a predominance of moderately differentiated tumors in 101 samples (82.8%), little differentiated in 10 (8.2%), well differentiated in eight (6.6%), and three (2.4 %) of undetermined. The most common site of metastasis at diagnosis was in the liver (n = 24, 19.7%), peritoneum (n = 9, 7.4%), lung (n = 5, 4.1%) and another organ (n = 11.9%)

Patients were classified according to their clinical stage, from 0 to IV, respecting the classification of the 6th edition of the Cancer Staging Manual (AJCC) of 2002, as used at the time when patients were seen (Table 1), with a predominance of the most advanced cases, stages III and IV with 74 cases (60.9%). 

**TABLE 1:** Distribution according to clinical stage

UICC	n	%
0	1	0,8
I	14	11,5
II	33	27
III	39	32
IV	35	28,7
Total	122	100

The ABCB5 control group was 14 (11.5%), case 1 of 74 patients (60.7%) and case 2 of 34 (27.8%), while in the OPN group the control was 53 (43, 4%), case 1 of 41 (33.6%) and case 2 of 28 (23.3%, Table 3). No statistical significance was observed in the presence or absence of the ABCB5 and OPN markers in relation to age, time of diagnosis of patients, gender, degree of differentiation, clinical staging or metastasis anywhere. The evaluation of the agreement between the ABCB5 and OPN markers (Table 2) was shown to be weak, with 24.6% of agreement in both positive (n = 30) and 5.7% (n = 7) in both negative.

**TABLE 2:** Concordance between markers

OPN	ABCB5			Total
	Negative	Positive	Inconclusive	
Negative	7	37	9	53
	5,70%	30,30%	7,40%	
Positive	6	30	5	41
	4,90%	24,60%	4,10%	
Inconclusive	1	7	20	28
	0,80%	5,70%	16,40%	
Total	14	74	34	122

Concurrent cases = 57 (46.7%); discordant cases = 65 (40.2%); *Kappa* coefficient of agreement: 0.22 (weak agreement)

The ABCB5 marker in cases of local disease was more associated with death in case 1 group [26/54 vs. 6/10; p = 0.161 (95% CI 0.21-1.29)], case group 2 [13/23 vs. 6/10; p = 0.631 (95% CI 0.3-2.08)] and patients with advanced disease, both in case 1 group [14/20 vs. 4/4; p = 0.509 (95% CI 0.22-2.13)] as for case group 2 [5/11 vs. 4/4; p = 0.122 (95% CI 0.09-1.32)], all of which were not statistically significant (Table 3). 

**TABLE 3:** Relationship of marker expression with clinical stage

ABCB5						
Variable	Classif	n	% of death	p*	HR	CI 95%
Stage 0/I/II	Negativo (ref)	10	6 (60,0)			
	Positivo	54	26 (48,1)	0,161	0,52	0,21 - 1,29
	Inconclusivo	23	13 (56,5)	0,631	0,79	0,30 - 2,08
Stage III/IV	Negativo (ref)	4	4 (100)			
	Positivo	20	14 (70,0)	0,509	0,68	0,22 - 2,13
	Inconclusivo	11	5 (45,4)	0,122	0,35	0,09 - 1,32

*Modelo de regressão de Cox e teste de Wald, p<0,05

**Table:** 

OPN						
Variable	Classif	n	% of death	p*	HR	CI 95%
Stage 0/I/II	Negativo (ref)	40	20 (50,0)			
	Positivo	29	14 (48,3)	0,844	0,93	0,47 - 1,86
	Inconclusivo	18	11 (61,1)	0,240	1,57	0,74 - 3,32
Stage III/IV	Negativo (ref)	13	10 (76,9)			
	Positivo	12	9 (75,0)	0,949	1,03	0,41 -2,60
	Inconclusivo	10	4 (40,0)	0,865	0,90	0,27 - 2,96

* Cox regression model and Wald test, p<0,05

Regarding the OPN marker, similar data were obtained in the local disease associated with the case 2 group, compared to the control group [20/40 vs. 11/18; p = 0.24 (95% CI 0.74-3.32)]. In patients with advanced disease, there was a higher death rate in the control group compared to the case 1 group [9/12 vs. 10/13; p = 0.949 (95% CI 0.41-2.6)] and case group 2 [4/10 vs. 10/13; p = 0.865 (95% CI 0.27-2.96)], all of which were not statistically significant (Table 3). 

When comparing groups with respect to disease progression of the ABCB5 marker, higher rates of progression occurred among patients in the case 1 group [30/74 vs. 2/14; p = 0.141 (95% CI 0.69-13.7)] and case group 2 [11/34 vs. 2/14; p = 0.229 (95% CI 0.54-12.8)], however did not result in statistically significant. Regarding the OPN marker, there were also higher rates in the case 1 group [16/41 vs. 17/53; p = 0.541 (95% CI 0.63-2.41)] and case group 2 [10/28 vs. 17/53; p = 0.404 (95% CI 0.64-3.03)], also not showing statistically significant results (Table 4). 

**TABLE 4:** Analysis of variables in relation to disease progression

Variable	Classification	n	% of cases with progression	p*	SHR	CI 95%
ABCB5	Negative (ref)	14	2 (14,3)			
	Positive	74	30 (40,5)	0,141	3,08	0,69 - 13,7
	Inconclusive	34	11 (32,2)	0,229	2,64	0,54 - 12,8
OPN	Negative (ref)	53	17 (32,1)			
	Positive	41	16 (39,0)	0,541	1,23	0,63 - 2,41
	Inconclusive	28	10 (35,7)	0,404	1,39	0,64 - 3,03

SHR = subdistribution hazard ratio; 95% CI = 95% confidence interval; * = Fine and Gray model and Wald test, p<0,05

In the assessment of factors associated with death for the marker ABCB5, a higher rate of evolution was observed in the control group in relation to case 1 [40/74 vs. 10/14; p = 0.089 (95% CI 0.27-1.1)] and case group 2 [18/34 vs. 10/14; p = 0.239 (95% CI 0.29-1.36)], both not statistically significant. Similar results were obtained in the OPN marker, with higher rates in the control group compared to the case 1 group [23/41 vs. 30/53; p = 0.982 (95% CI 0.58-1.74)] and case group 2 [15/28 vs. 30/53; p = 0.403 (95% CI 0.7-2.46)], without statistical significance (Table 5).

**TABELA 5:** Analysis of variables in relation to the death event

Variabel	Classification	n	% of death	p*	HR	CI 95%
ABCB5	Negative (ref)	14	10 (71,4)			
	Positive	74	40 (54,1)	0,089	0,54	0,27-1,10
	Inconclusive	34	18 (52,9)	0,239	0,63	0,29-1,36
OPN	Negative (ref)	53	30 (56,6)			
	Positive	41	23 (56,1)	0,982	1,01	0,58-1,74
	Inconclusive	28	15 (53,6)	0,403	1,31	0,70-2,46

*= Cox regression model and Wald test, p <0.05; Progression event variable was included as time-dependent

## DISCUSSION

Follow-up time was longer in stage II patients and shorter in stages 0 / I. This context can be explained by the loss of follow-up, the high cure rate, or even the loss of follow-up from the patient. The shortest follow-up periods were seen in stages III / IV, due to the natural evolution of the disease followed by death.

It is possible was raised that the levels of OPN in blood samples or tumor specimen could be valuable in predicting the prognosis of carcinomas. This fact motivated a meta-analysis to evaluate the expression of OPN both in progression and in prognosis, and its usefulness as a prognostic biomarker was verified, besides being a potential therapeutic target in RCC^32^. 

It was previously demonstrated that the high level of expression of OPN mRNA had clinicopathological significance and prognosis in RCC. The level of expression of the OPN protein in CCR cells and corresponding normal tissue samples was evaluated. Their results indicated that the negative regulation of OPN could suppress both in vitro proliferation and in vivo tumorigenicity^15^. The expression of OPN in a CCR cell in this study was positive in 39% of cases of progression and 56.1% of deaths. However, this data was not statistically significant.

In a study with 84 CRC patients, it was found that the level of transcription of OPN and its overexpression were responsible for inducing chemo-resistance to treatment with oxaliplatin. This overexpression was related to metastasis and decreased survival rate^27^. In this sample, a rate of 39% of disease progression and 56.1% of cases of death were observed, which, despite not having statistical significance, is believed to be related to the more advanced cases and which presented some type of chemoresistance to the treatment offered. . In 59.7% (46/77) of the times when chemotherapy was a treatment option, oxaliplatin was provided both in neoadjuvancy and in adjuvance and palliation.

In a group of more than 200 CCR stage II patients, it was noted that the level of OPN in the tumor tissue was useful in the detection of CCR, but it was not related to prognosis^21^. In this research, 33 stage II cases (27%) were observed, of which 10 (30.3%) presented disease progression and 15 (45.4%) progressed to death. Despite the absence of statistical significance, these are numbers that cannot be ignored. 

Likui et al. reported that the level of OPN mRNA expression in cells or tissues with RCC was significantly higher than in non-tumoral epithelial cells of the colon^15^ ^,^ ^18^. It has also been reported that the value of OPN mRNA expression in the CCR enhances clinicopathological and / or prognostic findings. In addition, it was shown that the level of OPN mRNA expression in RCC cells was significantly associated with lymph node metastasis, lymphatic invasion, venous invasion and consequently TNM staging (advanced stages - III / IV)^16^. This increase in OPN expression was observed in this research, with a predominance in cases of local disease (stage 0 / I / II), but without statistical significance.

When analyzing the data related to the presence of the ABCB5 marker, its positivity was identified in 60.7% (n = 74) of the cases of a total of 122 patients. Despite this high incidence of positive ABCB5 expression alone, there was poor corelation between ABCB5 and OPN, with only 24.6% of positive cases.

The present sample did not achieve statistical significance for the epidemiological data related to ABCB5; however, there was a correlation between positivity of this marker with a diagnosis of CRC and death. When the positive relationship between ABCB5 and local or advanced disease was assessed, there was a greater positivity of the marker with stage III and IV (more advanced disease), again without statistical significance. Two possibilities were raised by Kugimiya et al. which help to justify this finding: the first would be the survival of CCR cells treated with 5-FU and which showed high ABCB5 expression; the other would be that the use of 5-FU increases the expression of the marker in some cells, and with that they can stay alive^13^. 

It was found that in cases that required chemotherapy, 100% were treated with fluoropyrimidine at some point, and showed 40.5% disease progression and 54.1% death. There was agreement in the findings above and in the literature, especially in situations in which the marker functions as a mediator of resistance to multiple drugs, such as 5-FU^10^, and especially when used as monotherapy^29^.

## CONCLUSION

The immunohistochemical expression of the OPN and ABCB5 markers was not associated with epidemiological and clinicopathological characteristics. Regarding the progression of the disease and death, it was not possible to observe a correspondence relationship with the evaluated markers.
